# Unmanned Aerial Vehicle in the Logistics of Pandemic Vaccination: An Exact Analytical Approach for Any Number of Vaccination Centres

**DOI:** 10.3390/healthcare10102102

**Published:** 2022-10-20

**Authors:** Adnan Benayad, Olaf Malasse, Hicham Belhadaoui, Noureddine Benayad

**Affiliations:** 1 Laboratory of Computer Networks, Telecommunications and Multimedia, ESTC, Hassan II University, B.P. 8012 Oasis, Casablanca 20000, Morocco; 2 National School of Electricity and Mechanics, Hassan II University, B.P. 8118 Oasis, Casablanca 20000, Morocco; 3 Laboratoire LCFC, National School of Arts and Crafts, ParisTech, 57070 Metz, France; 4 Laboratory of PHEMaC, Faculty of Sciences-Ain Chock, Hassan II University, B.P. 5366 Maarif, Casablanca 20000, Morocco

**Keywords:** pandemic vaccination, exact analytical approach, unmanned aerial vehicle (UAV), drone delivery, route planning problem, optimal flight path, equivalent graphs, degeneracy

## Abstract

While the development and manufacture of pandemic vaccines is a daunting task, the greatest challenge lies in how to deliver these vaccines to billions of people around the world. This requires an efficient strategy of deliveries, at constrained costs and deadlines. This paper proposes an exact analytical approach and operational strategy to the logistics of any pandemic vaccination efforts, applicable both to sparsely populated areas or deficient infrastructure, and to very dense urban fabrics where mobility is highly constrained. Our strategy consists in dividing the territory concerned into zones and districts in a concentric way. We opt for the use of unmanned aerial vehicles to free ourselves from land constraints. This involves serving, from a logistics centre (central depot), any number n of vaccination centres, while optimizing costs and deadlines. We have determined all equivalent and optimal flight path plans for a fixed and optimal number of drones, which depend on domain D(d); d being the demand of vaccination centers. The analysis of the results led us to define what we will call the “degeneracy of domain D”. All our results are expressed as a function of the parameter n.

## 1. Introduction

Until recently, Unmanned Aerial Vehicles (UAVs) or drones have primarily been used in the military. Recently, they are becoming more present in many civilian sectors. This will open new possibilities for further research and development of UAVs. They are considered as one of the technological innovations which may trigger a revolutionary reshaping of transportation industry, since they have the potential to significantly reduce the cost and time required to deliver packages. By performing these tasks autonomously, drones may be faster than traditional delivery vehicles such as trucks since they are not limited by established infrastructure such as roads, and generally face less complex obstacle avoidance scenarios which complies with current trends in the transport industry [1].

It is noteworthy that a lot of pilot projects have been launched to exploit the potentials of drones in logistics applications. Examples of large organisations experimenting with drones are Google, DHL, and Amazon Indeed, in 2013, Amazon announced Prime Air [2,3], a service that utilizes multirotor drones to deliver packages from Amazon to customers. German logistics company Deutsche Post DHL also started its Parcelcopter project in 2013; the Parcelcopter has transported medicine to the island of Juist in the North Sea [4]. Google revealed Project Wing in 2014 to produce drones that can deliver larger items than Prime Air and Parcelcopter [5]. A startup called Matternet has partnered with Swiss Post to test a lightweight package delivery quadcopter [6]. Next to large-scaled projects of multi-national firms, also smaller-scaled projects of drone delivery systems have been successfully put into practice [7].

Non-military UAVs come in various shapes and sizes and typically contain a main airframe, navigation system and propulsion systems [8]. In medicine we use two primary types (i) fixed-wing aircraft and (ii) helicopter-like drones with single or multiple rotors.

For logistical applications, the speed, payload capacity and radius of operation are the most important technical parameters. They vary greatly among different drone models. Primarily models with payload capacity up to 5 kg are used [9,10]. However, also heavy-load UAVs are available with a payload capacity of up to 40 kg [11]. Drones, such as those produced by Zipline (Half Moon Bay, CA, USA), can fly at a speed of up to 128 km/h (80 mph) and have a range of 160 km (99 miles) round trip [12].

Developments in numerous technologies have enabled the above organizations to improve drone deliveries. Carbon fibre manufacturing costs have decreased significantly over the last years [13,14], enabling the development of strong, lightweight airframes. Lithium polymer batteries, known by their relatively high energy density [15] have improved the flight times of the drones compared to alternative technologies such as nickel-cadmium and nickel-metal hydride. UAVs typically use GPS to determine their location, and they are also able to take advantage of DGPS and localization techniques [16,17] to improve accuracy. Obstacles can be avoided through many techniques such as LIDAR and image processing [18,19]. Architectures and protocols have been developed that enable drones to form ad-hoc networks and to wirelessly communicate with other entities [20,21].

Other technological issues to be considered in logistical UAV applications are the launching and landing concept in addition to the autonomous control capability. Meanwhile, most commercial UAV models also provide fully automatic launching stations [22]. For B2C concepts, however, the UAV must be able to land on ‘rough’ ground. Further, some detachment technology like ropes (Flirty, Google) or parachutes (Zipline) could be available. In a B2C application scenario, it is reasonable to assume that UAVs must wait hovering until all prerequisites for detaching the cargo are fulfilled (e.g., waiting for the customer’s approval or a clear detachment area).

Drones’ systems have also been reported in other practical applications in emergency and disaster management situations where the crucial feature is the drone’s ability to travel directly between several points of interest [23] over hazardous terrain during a crisis. These include intelligence, surveillance and reconnaissance (ISR) missions to visit a n set of locations [24]; and in emergency aid in order to reduce the worker’s exposure to danger and also for emergency response in the event of forest fires, oil spills, and earthquakes [25]. UAVs equipped with cameras allow for viewing disaster scenes promptly, collecting critical data including aerial photograph, air quality or radiation levels. They can deploy wireless sensors to provide immediate updates on the event to the teams on the ground. On the other hand, in the healthcare sector, especially in developing countries [26], UAVs have already been used in different aspects, including transfer of blood product. They can also be used to transport diagnostic samples and various medical purposes [8,27]. Finally, we note that such drones are also used in agriculture (monitoring crop production), construction (surveying land), industry (warehouse management), public safety (law enforcement and traffic surveillance), and environmental conservation efforts (deforestation monitoring),

In a world where logistics has become a vital function, as we were able to verify during the COVID-19 health crisis, but where the margins of the various players in the supply chain are increasingly tight and prices constantly drawn down, the drone is a solution to consider and seriously study for all those who want to increase their operational efficiency and stand out for their quality of service.

The purpose of the paper is to offer an exact analytical approach and operational strategy to the logistics of any pandemic vaccination effort. Our study gives an answer to the following question: How to use drones in order to deliver pandemic vaccines to large areas (densely populated city or sparsely populated rural region) whatever the number of the population living there. Indeed, this paper primarily studies the route planning problem of the UAVs during distribution. The application scenario is the vaccines delivery from the distribution centre to the vaccination centres, and to determine all equivalent and optimum flight path plans for drones that need to serve multiple positions for any number of vaccination centres.

The structure of this paper is as follows. Section 2 provides a review on the relevant literature. In Section 3, we formally introduce the strategy, hypotheses and used notations. Section 4 describes the drone routing problem for fixed numbers of vaccination centres. In Section 5, we provide a generalization of the drone routing problem, i.e., for any number of vaccination centres. This problem leads us to define what can be called “the degree of degeneracy” of the vaccination centres demand. Section 6 concludes the paper.

## 2. Literature Review: Use of Drones in Healthcare

Research on employing drones in delivery operations has gained a lot of attention in recent years. There is an exploding body of literature on potential application scenarios concerning this subject. An extensive overview about civil applications of UAVs can be found in reference [28].

Drones have been used in several sectors of healthcare. Preliminary reports have indicated the feasibility of drone related transfer of biological samples (for instance, blood product) during short flights at room temperatures or colder with no significant influence on the accuracy of routine chemistry, haematology and coagulation analyses [29,30]. In this sector, we must notice that Rwanda was the first country to successfully use drones into health services at the national level. A drone delivery programme also known as ‘Uber for blood’ was launched in 2016. It uses battery-powered fixed-wing drones designed and built by Zipline capable of flying up to 150 km in a round trip and carrying up to 1.5 kg of blood. We note here that Rwandan patients have never received blood quickly and so efficiently: Indeed, blood delivery times have plummeted from approximately 4 h to only 15–45 min in remote areas. More than 18,000 life-saving delivery flights containing blood products were carried out in August 2019 [31].

In 2016, UNICEF and the Government of Malawi initiated an important programme in order to explore whether sample transportation by UAV is a cost-effective intervention to reduce time-to-result for human immunodeficiency virus testing in infants [32]. Drone have been also used in another sector of healthcare. In Papua New Guinea, where the prevalence of tuberculosis is one of the highest in the world (nearly 6/1000 population/year), drones were used to transport sputum samples of individuals with suspected tuberculosis from dispersed health centres to Kerema General Hospital, which circumvented the need to use road transport that was hampered during the rainy months [33].

On the hand, in 2017, Switzerland paved the way for the transport of specimens by drone in Europe by authorizing flights of autonomous drones for healthcare services over cities at any time. Swiss Post and Matternet have developed a medical transport network using quadcopter drones (20-km range, average speed 36 km/h, 2-kg maximum payload), with more than 3000 successful flights in Lugano, Bern and Zurich in April 2019 [27,34].

A drone programme has also been successfully implemented in Tanzania, a country with one of the highest maternal mortality rates in the world (556 deaths/100 000 deliveries). The drones were much faster than ground transportation, delivering on-demand blood, vaccines, and antiretroviral and malaria drugs via biodegradable parachutes to more than 1000 health facilities [35].

In April 2019, Gavi, the Vaccine Alliance, announced the launch of the largest drone healthcare project. Delivery of blood, medicines and vaccines is now available for 2000 health facilities serving 12 million people across Ghana. Distribution centres can deliver up to 600 on-demand drone transports per day with potential for further expansion to up to 2000 flights/day [36].

The economic and operational value concerning vaccine deliveries was recently assessed using a computational model [7]. Compared with traditional land transport, drone delivery increased vaccine availability and decreased costs ($0.05 to $0.21 per dose administered), proving that drones are cost-effective and useful in a variety of circumstances and settings if used frequently enough to overcome the system installation and maintenance costs [37]. We note that drone delivery has been successfully piloted in Vanuatu (a Pacific archipelago) where most villages are not easily reachable and often have no electricity to store vaccines, leaving nearly 20% of Vanuatu’s 35,000 children under 5 years not fully vaccinated [37].

The World Health Organization has designated COVID-19 as a pandemic. Currently, over 140 million cases of COVID-19 have been confirmed worldwide, including more than 3,000,000 deaths. There is a dire need for therapeutics and vaccines to fight such pandemic. Distributing an effective vaccine to billions of people around the world is likely the greatest logistical challenge since the Second World War. First, to satisfy the high-demands during the COVID-19, some models have been developed [38] in order to increase the production of vaccines. On the other hand, they should be transported, from the manufacturing sites to distributors. These latter must deliver the vaccines to vaccination centres, which have to be adequately and uniformly distributed in urban and rural areas. This is not an easy task, especially if we know that maintaining the cold chain will be a crucial issue for such vaccines, which poses a significant problem in many parts of the world. Low temperatures must be maintained whether vaccines are being delivered to densely populated cities or sparsely populated rural areas. We must note that the pandemic vaccines have to be stored within appropriate and efficient conditions. This is the health guidelines state that even small deviations can render a vaccine ineffective. According to a 2019 report by the International Air Transport Association (IATA), approximately 25% of vaccines shipped are at risk due to poor temperature management in transportation vehicles. The report estimates that the associated damage costs the healthcare industry more than $34 billion annually. For the people and economies that depend on the prevention of COVID-19, inefficient cold chain management will be particularly costly. Thus, healthcare distributors and providers must operate in a specialized, temperature-controlled supply chain. It is worthy to note that in the context of the COVID-19 pandemic that Zipline is deploying its ingenious logistics. Faced with problems relatively identical to those of Rwanda, the Ghanaian government has also chosen to call on the Californian start-up for the delivery, initially of tests, then now of Covid vaccines. About 2.5 million doses are expected to be delivered in Ghana using these drones [31]. Not only does this make Ghana the first country in the world to deploy drones nationwide for the delivery of COVID-19 vaccines, but it is also a mammoth effort to ensure equitable access and enable Ghana to use fully its healthcare infrastructure to deliver vaccines,” Zipline CEO Keller Rinaudo said in a statement.

## 3. Strategy, Hypotheses and Notations

### 3.1. Strategy

**Figure 1:**
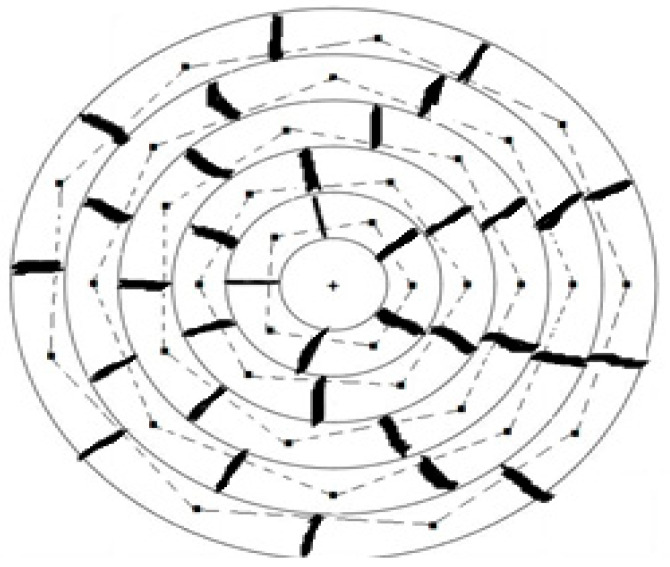
Localization of all vaccination centres belonging to the concerned territorial area.

In this paper, we are interested in the use of drones in logistics of any pandemic vaccination. The strategy we adopt to vaccinate the population of a given territorial area (urban or rural) is clearly shown in Figure 1. Indeed,

(i) We divide the territorial area into M zones constituting M concentric circular bands with the same width.
(ii) Each band is divided into n isometric districts where the centre of each one of them is chosen as the vaccination center Thus, the set of vaccination centres in a given zone, are located on the vertices of a regular n-polygon.
(iii) Each vaccination centre deals with the vaccination of people living in its own district.
(iv) All vaccination centres belonging to the same zone (circular band, see Figure 1), are served independently from the others.

### 3.2. Hypotheses

In order to perform such vaccination operation using drones, we have to study the route planning problem of the UAVs distribution. To this end, we consider the following assumptions:

1- The drones’ platform, namely the starting and finishing points of the drones, is located on the centre of the considered territorial area which coincides with the common circumcenter of the circumcircles of regular polygons locating all the vaccination centres.
2- The drone is a single model. So, the load capacity is the same and fixed.
3- The drone batteries are fully charged before leaving the platform. The charge is enough to satisfy the maximum centres, according to the drone load capacity.
4- The vaccination centres make the same demands.
5- The vaccines are stored within appropriate and efficient conditions during the transportation.
6- Each trip is composed of segments (edges and radius of the n-polygon) interconnecting the locations, measured by the Euclidean distance between them. They are crossed at constant speed by the drone which is independent of its load capacity.
7- Each vaccination centre must be served only by one drone.
8- For a given range of vaccination centres demands, only optimum numbers of drones are used to cover the entire demands.
9- For a given range of vaccination centres demands, there is at least one drone that satisfies a maximum number of centres.

### 3.3. Notation

We define the different sets needed for modelling the problem in addition to the parameters and variables.
MNumber of zones (circular bands) covering the entire territorial area concerned by vaccination.nnumber of vaccination centres belonging to the same zone.dQuantity of vaccines demanded by vaccination centre.CLoad capacity of the droneNoptimum number of drones used for the logistic distribution.(k_1_, k_2_, …, k_N_)/k_1_
≥ k_2_
≥_,_ …, ≥ k_N_, with $\overset{N}{\underset{i=1}{\sum }}k_{i}=n$, where k_i_ is the number of vaccinations centres visited by the *i*th drone.k_1_(d)denotes the maximum number of vaccination centres that one drone can serve, according to the domain D of d.G = {n, N, (k_1_, k_2_, …, k_N_)}Graph defining the set of paths followed by the N drones in order to deliver vaccines to the n vaccinations centres belonging the same zone. For instance, the path described by the *i*th drone is formed by the set of locations or vertices to be visited and the set of directed links connecting them. 2 rThe radius of the first zone. It represents also the width of circular bands. 

## 4. Drone Routing Problem for Fixed Number of Vaccination Centres

### 4.1. Optimum Number of Drones and Vaccination Centre Demands

Before giving our general formulation of logistics distribution for any number n of vaccination centres and valid for any zone, we give in this section, a detailed presentation of the model in the case of small, fixed values of n = 5,6,7,8, and 9.

First, we represent the correlation between the optimum number of drones necessary to use for logistics distribution and the needs of the vaccination centres. The cases n = 7,8, and 9 are presented in Table 1. The other cases corresponding to n = 5 and 6, are presented in Appendix A.

In the first column, we have considered all possible ratios d/C between the demand d of each vaccination centre and the load capacity C of the drone. In the second column, we indicate the maximum number k_1_(d) of vaccination centres that one drone can serve. In the third column, we note optimum number N of drone we should use for each logistics distribution, according to the various ranges of vaccination centres demands. In the fourth column, we represent the exact graphs calculated in the frame of our approach.

In all Tables, we represent the length *L* (*n, N*) of the paths travelled by the set of the optimal number of drones necessary for logistics distribution. Its general expression for any n and N is given by the following relation:

(1) $$L\left(n,N\right)=\left(n-N\right)a_{n}+2Nr_{n}$$

where $a_{n}$ is the distance between two nearest vaccination centres belonging to the same n-polygon (zone) and $r_{n}$ is its circumradius. They are given by:

$$a_{n}=2\left(2n-7\right)sin\left(\frac{\pi }{n}\right)r$$

$$\;r_{n}=\left(2n-7\right)r$$

It is important to notice that this length is constant for any fixed N and therefore it does not depend on the corresponding intervals, although these latter correspond to various graphs.

**Figure 2:**
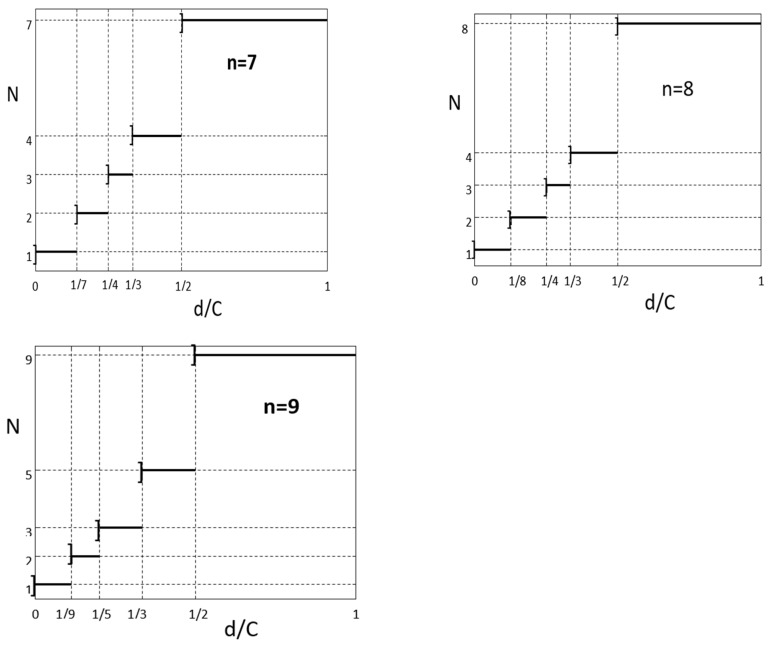
The variation of optimum number N of drones as function of vaccination centre demands, for n = 7, 8, and 9.

In order to give an elegant formulation of the results demonstrated in the above Tables, we represent in Figure 2, the variation of optimum number N of drones as function of vaccination centre demands. This is done for different number n of centres. As is observed in these figures, this variation can be described by a function which can be called “ceiling function with unequal steps”.

The other cases corresponding to n = 5 and 6 are presented in Appendix A.

### 4.2. Degeneracy of the Domains of Vaccination Centres Demands

In the rest of our investigation, we are interested in the study of the domains (intervals) of d/C where the set of vaccination centre demands, belonging to the same zone, are served by a fixed optimum number N of drones.

It is worthy to notice an interesting behaviour related to the topology of the graphs. It concerns the existence of multiple graphs for a defined range of vaccination centre demands satisfying a well-defined maximum number of centres. For instance, this is observed in the case N = 3 for n = 7 and 9 when d/C belongs to ranges $\left[\frac{1}{4},\frac{1}{3}\right]$ and $\left[\frac{1}{5},\frac{1}{4}\right]$, respectively. The corresponding variety of graphs can be called “equivalent graphs” in the sense that they have the same length of paths taken by the N drones but different topologies. Therefore, they use the same energy consumption during logistics distribution. This behaviour can be called the degeneracy of the corresponding domain. In the present paper, we focus our study to two cases, namely: (i) N = 2 and (ii) N = 3.

**Figure 3:**
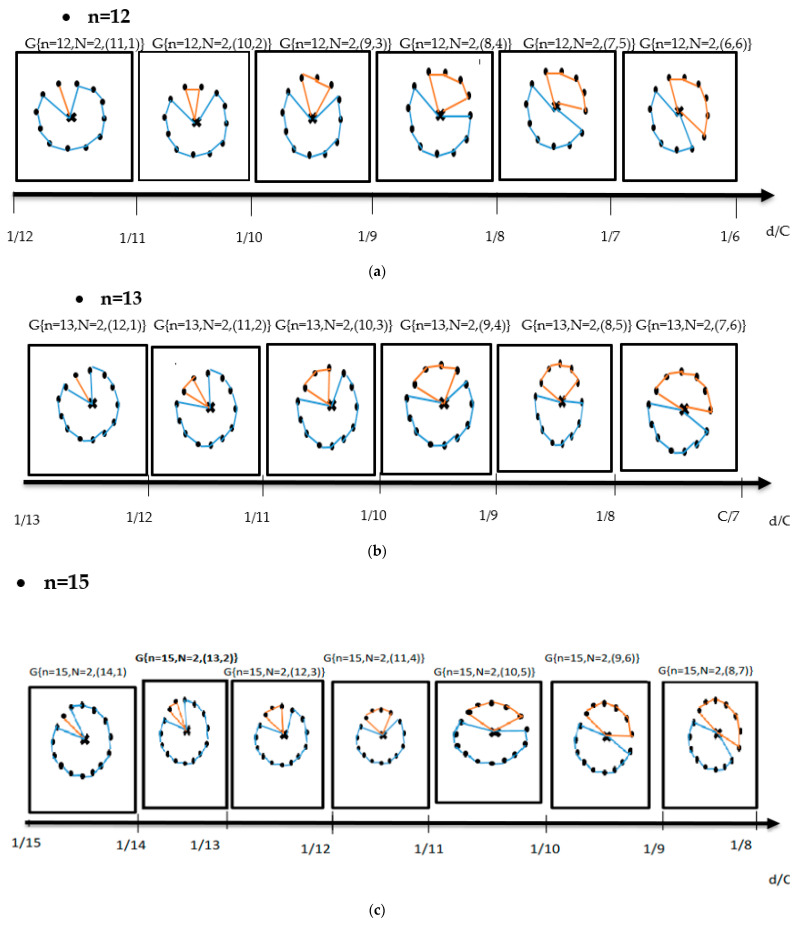
Set of possible graphs corresponding to the various domains of d/C for N = 2, (**a**) n = 12, (**b**) n = 13, and (**c**) n = 15.

**Figure 4:**
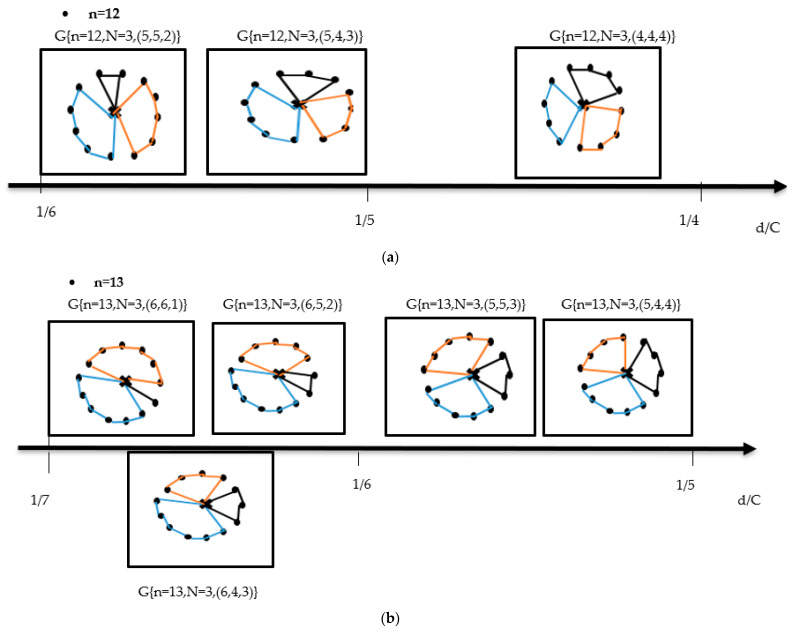

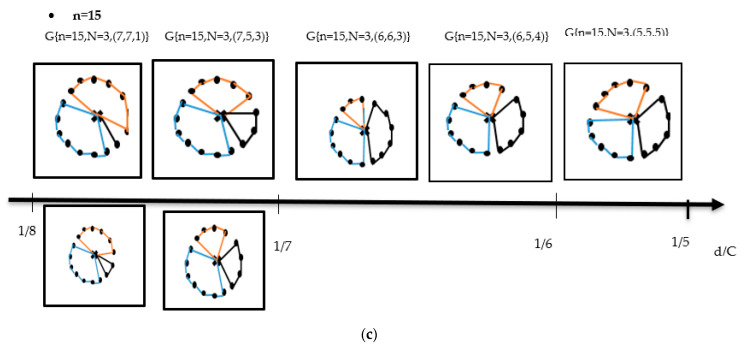
Set of possible graphs corresponding to the various domains of d/C for N = 3, (**a**) n = 12, (**b**) n = 13, and (**c**) n = 15.

In Figure 3 and Figure 4, we draw for various zones which contain respectively different centres (n = 12, 13, and 15), the set of possible graphs corresponding to the various domains D of d/C.

(i) For N = 2, we obtain

As is observed from Figure 3, we note that, in every range of d/c, there is one and only one graph. We note that all graphs corresponding to N = 2 have different topologies which depend on the maximum number of vaccination centres that can be satisfied by one drone.

(ii) For N = 3, we obtain

In Figure 4, we represent all possible graphs for N = 3, according to each domain of vaccination centre demands. We note the existence of multitude graphs in well-defined domains of d/C. For instance, in the case (n = 15, N = 3), when d/C belongs to $\frac{1}{8}<\frac{d}{C}\leq \frac{1}{7}$, we have 4 equivalent graphs which they have the same global length and therefore the same “global” cost and the same delivery time.

In order to give a precise description to the above interesting behaviour, we suggest a new nomenclature describing this variety of equivalent graphs. To this end, we introduce the following definition: We define the degeneracy of a domain D$\left(\alpha <\frac{d}{C}\leq \beta \right)$ by the number of equivalent graphs existing in the specified range $\left(\alpha <\frac{d}{C}\leq \beta \right)$. In addition to the degeneracies expressed in Figure 4, we have calculated the degeneracy of domains corresponding to N = 3 for zones containing large numbers of vaccination centres (n = 18, 21, 24, and 27). Below, we give their degrees and corresponding graphs.
Deg(n = 18, N = 3, D$\left(\frac{1}{9}<\frac{d}{C}\leq \frac{1}{8}\right)$) = 4, G{n = 18, N = 3, (8, 8, 2)}, G{n = 18, N = 3, (8, 7, 3)},
G{n = 18, N = 3, (8, 6, 4)},G{n = 18, N = 3, (8, 5, 5)}.Deg(n = 18, N = 3, D$\left(\frac{1}{8}<\frac{d}{C}\leq \frac{1}{7}\right)$) = 2, G{n = 18, N = 3, (7, 7, 4)}, G{n = 18, N = 3, (7, 6, 5)}.Deg(n = 18, N = 3, D$\left(\frac{1}{7}<\frac{d}{C}\leq \frac{1}{6}\right)$) = 1, G{n = 18, N = 3, (6, 6, 6)}.Deg(n = 21, N = 3, D$\left(\frac{1}{11}<\frac{d}{C}\leq \frac{1}{10}\right)$) = 5, G{n = 21, N = 3, (10, 10, 1)}, G{n = 21, N = 3, (10, 9, 2)},
G{n = 21, N = 3, (10, 8, 3)}, G{n = 21, N = 3, (10, 7, 4)},
G{n = 21, N = 3, (10, 6, 5)}.Deg(n = 21, N = 3, D$\left(\frac{1}{10}<\frac{d}{C}\leq \frac{1}{9}\right)$) = 4, G{n = 21, N = 3, (9, 9, 3)}, G{n = 21, N = 3, (9, 8, 4)},
G{n = 21, N = 3, (9, 7, 5)}, G{n = 21, N = 3, (9, 6, 6)}Deg(n = 21, N = 3, D$\left(\frac{1}{9}<\frac{d}{C}\leq \frac{1}{8}\right)$) = 2, G{n = 21, N = 3, (8, 8, 5)}, G{n = 21, N = 3, (8, 7, 6)}.Deg(n = 21, N = 3, D$\left(\frac{1}{8}<\frac{d}{C}\leq \frac{1}{7}\right)$) = 1, G{n = 21, N = 3, (7, 7, 7)}.Deg(n =24, N = 3, D$\left(\frac{1}{12}<\frac{d}{C}\leq \frac{1}{11}\right)$) = 5, G{n = 24, N = 3, (11, 11, 2)}, G{n = 24, N = 3, (11, 10, 3)},
G{n = 24, N = 3, (11, 9, 4)}, G{n = 24, N = 3, (11, 8, 5)},
G{n = 24, N = 3, (11, 7, 6)}.Deg(n = 24, N = 3, D$\left(\frac{1}{11}<\frac{d}{C}\leq \frac{1}{10}\right)$) = 4, G{n = 24, N = 3, (10, 10, 4)}, G{n = 24, N = 3, (10, 9, 5)},
G{n = 24, N = 3, (10, 8, 6)}, G{n = 24, N = 3, (10, 7, 7)}Deg(n = 24, N = 3, D$\left(\frac{1}{10}<\frac{d}{C}\leq \frac{1}{9}\right)$) = 2, G{n = 24, N = 3, (9, 9, 6)}, G{n = 24, N = 3, (9, 8, 7)}.Deg(n = 24, N = 3, D$\left(\frac{1}{9}<\frac{d}{C}\leq \frac{1}{8}\right)$) = 1, G{n = 24, N = 3, (8, 8, 8)}.Deg(n =27, N = 3, D$\left(\frac{1}{14}<\frac{d}{C}\leq \frac{1}{13}\right)$) = 7, G{n = 27, N = 3, (13, 13, 1)}, G{n = 27, N = 3, (13, 12, 2)},
G{n = 27, N = 3, (13, 11, 3)},G{n = 27, N = 3, (13, 10, 4)},
G{n = 27, N = 3, (13, 9, 5)}, G{n = 27, N = 3, (13, 8, 6)},
G{n = 27, N = 3, (13, 7, 7)}.Deg(n=27, N = 3, D$\left(\frac{1}{13}<\frac{d}{C}\leq \frac{1}{12}\right)$) = 5, G{n = 27, N = 3, (12, 12, 3)}, G{n = 27, N = 3, (12, 11, 4)},
G{n = 27, N = 3, (12, 10, 5)}, G{n = 27, N = 3, (12, 9, 6)},
G{n = 27, N = 3, (12, 8, 7)}.Deg(n= 27, N = 3, D$\left(\frac{1}{12}<\frac{d}{C}\leq \frac{1}{11}\right)$) = 4, G{n = 27, N = 3, (11, 11, 5)}, G{n = 27, N = 3, (11, 10, 6)},
G{n = 27, N = 3, (11, 9, 7)}, G{n = 27, N = 3, (11, 8, 8)}.Deg(n = 27, N = 3, D$\left(\frac{1}{11}<\frac{d}{C}\leq \frac{1}{10}\right)$) = 2, G{n = 2, N = 3, (10, 10, 7)}, G{n = 27, N = 3, (10, 9, 8)}.Deg(n = 27, N = 3, D$\left(\frac{1}{10}<\frac{d}{C}\leq \frac{1}{9}\right)$) = 1, G{n = 27, N = 3, (9, 9, 9)}.

## 5. Drone Routing Problem for Any Number of Vaccination Centres

In this section, we reformulate our model for any number (n ∈ IN) of vaccination centres. The optimum number of drones that can be used to perform the distribution is N = 2 and 3.

Consider a zone and let n be the number of vaccination centres located on the sites (vertices) of the regular n-polygon inscribed in that zone. The drone’s platform is situated on the centre of the circumcircle of the polygon.

### 5.1. General Expressions of Demand Domains and Their Corresponding Graphs

First, let us find for any fixed n, the set of domains D ($\alpha <\frac{d}{C}\leq \beta )$ defined in the previous section. The domains concerned by our generalization are those where the vaccination centres are served by an optimal number of drones equal to 3 (N = 3). To this end, it should be noted that these domains depend on the parity of n.

(a) For odd numbers n ≥ 3

The possible domains and corresponding graphs are given by:
Domains: DEquivalent graphsD_0_($\frac{1}{\frac{n+1}{2}}<\frac{d}{C}\leq \frac{1}{\frac{n-1}{2}})$
G{n, N = 3, ($\frac{n-1}{2}$,$\frac{n-1}{2},\;1)$}$k_{1}\left(d\right)=\frac{n-1}{2}$G{n, N = 3, ($\frac{n-1}{2}$,$\frac{n-3}{2},\;2)$}
**|**, with m = {$\begin{array}{c} \frac{n}{4}-\frac{3}{4}\;,\;n\in \left\{3+4k/k\in IN\right\}\\ \frac{n}{4}-\frac{5}{4}\;,\;n\in \left\{5+4k/k\in IN\right\} \end{array}$
G{n, N = 3, ($\frac{n-1}{2}$,$\frac{n-1}{2}-m,\;1+m)$}D_1_($\frac{1}{\frac{n-1}{2}}<\frac{d}{C}\leq \frac{1}{\frac{n-3}{2}})$
G{n, N = 3, ($\frac{n-3}{2}$,$\frac{n-3}{2},\;3)$}$k_{1}\left(d\right)=\frac{n-3}{2}$G{n, N = 3, ($\frac{n-3}{2}$,$\frac{n-5}{2},\;4)$}
**|**, with m = {$\begin{array}{c} \frac{n}{4}-\frac{9}{4}\;,\;\;n\in \left\{9+4k\;/k\in IN\right\}\\ \frac{n}{4}-\frac{11}{4}\;,\;\;n\in \left\{11+4k\;/k\in IN\right\} \end{array}$
G{n, N = 3, ($\frac{n-3}{2}$,$\frac{n-3}{2}-m,\;3+m)$}D_2_($\frac{1}{\frac{n-3}{2}}<\frac{d}{C}\leq \frac{1}{\frac{n-5}{2}})$
G{n, N = 3, ($\frac{n-5}{2}$,$\frac{n-5}{2},\;5)$}$k_{1}\left(d\right)=\frac{n-5}{2}$G{n, N = 3, ($\frac{n-5}{2}$,$\frac{n-7}{2},\;6)$}
**|**, with m = {$\begin{array}{c} \frac{n}{4}-\frac{15}{4}\;,\;\;n\in \left\{15+4k\;/k\in IN\right\}\\ \frac{n}{4}-\frac{17}{4}\;,\;\;n\in \left\{17+4k\;/k\in IN\right\} \end{array}$
G{n, N =3, ($\frac{n-5}{2}$,$\frac{n-5}{2}-m,\;5+m)$}D_3_($\frac{1}{\frac{n-5}{2}}<\frac{d}{C}\leq \frac{1}{\frac{n-7}{2}})$
G{n, N = 3, ($\frac{n-7}{2}$,$\frac{n-7}{2},\;7)$}$k_{1}\left(d\right)=\frac{n-7}{2}$G{n, N = 3, ($\frac{n-7}{2}$,$\frac{n-9}{2},\;8)$}
**|**, with m = {$\begin{array}{c} \frac{n}{4}-\frac{21}{4}\;,\;\;n\in \left\{21+4k\;/k\in IN\right\}\\ \frac{n}{4}-\frac{23}{4}\;,\;\;n\in \left\{23+4k\;/k\in IN\right\} \end{array}$
G{n, N = 3, ($\frac{n-7}{2}$,$\frac{n-7}{2}-m,\;7+m)$}D_*p*_($\frac{1}{\frac{n-1}{2}-p+1}<\frac{d}{C}\leq \frac{1}{\frac{n-1}{2}-p})$
G{n, N = 3, ($\frac{n-1}{2}-p$,$\frac{n-1}{2}-p,\;2p+1)$}$k_{1}\left(d\right)=\frac{n-1}{2}-p$G{n, N = 3, ($\frac{n-1}{2}-p$,$\frac{n-3}{2}-p-1,\;2p+2)$}
**|**, with m = {$\begin{array}{c} \frac{n}{4}-\frac{3\left(2p+1\right)}{4}\;,\;\;n\in \left\{3+6p+4k\;/k\in IN\right\}\\ \frac{n}{4}-\frac{3\left(2p+1\right)}{4}-\frac{1}{2},\;n\in \left\{5+6p+4k\;/k\in IN\right\} \end{array}$
G{n, N = 3, ($\frac{n-1}{2}-p$,$\frac{n-1}{2}-p-m,\;2p+1+m)$}where *p* is the smallest integer value satisfying the following inequality:

$$3\left(\frac{n-1}{2}-p-1\right)<n.$$

It follows that *p* is given by:

(2) $$p=floor\left(\frac{n-9}{6}\right)+1$$

which can be expressed by the following expression:

$$p=\left\{\begin{array}{c} \frac{n-9}{6}-\frac{1}{\pi }arccot\left[cot\left(\frac{n-9}{6}\pi \right)\right]+1,\;n\neq 12k+15/k\in IN\\ \frac{n-9}{6}+1,\;\;\;\;\;\;\;\;\;\;\;\;\;\;\;\;\;\;\;\;\;\;\;\;\;\;\;\;\;\;\;\;\;\;\;\;\;\;\;\;\;\;\;\;\;n=12k+15/k\in IN \end{array}\right\}$$

(b) For even numbers n ≥ 6

We note that for n = 2 or 4, there is no domains and equivalent graphs corresponding to N = 3. For even numbers n ≥ 6, the possible domains $\bar{D\;}$ and corresponding graphs are given by:
Domains: $\bar{D\;}$
Equivalent graphs${\bar{D}}_{1}$($\frac{1}{\frac{n}{2}}<\frac{d}{C}\leq \frac{1}{\frac{n}{2}-1})$G{n, N = 3, ($\frac{n}{2}-1$,$\frac{n}{2}-1,\;2)$}$k_{1}\left(d\right)=\frac{n}{2}-1$G{n, N = 3, ($\frac{n}{2}-1$,$\frac{n}{2}-2,\;3)$}
**|**, with m = {$\begin{array}{c} \frac{n}{4}-\frac{3}{2},\;n\in \left\{6+4k\;/k\in IN\right\}\\ \frac{n}{4}-2,\;n\in \left\{8+4k\;/k\in IN\right\} \end{array}$
G{n, N = 3, ($\frac{n}{2}-1$,$\frac{n}{2}-1-m,\;2+m)$}${\bar{D}}_{2}$($\frac{1}{\frac{n}{2}-1}<\frac{d}{C}\leq \frac{1}{\frac{n}{2}-2})$G{n, N = 3, ($\frac{n}{2}-2$,$\frac{n}{2}-2,\;4)$}$k_{1}\left(d\right)=\frac{n}{2}-2$G{n, N = 3, ($\frac{n}{2}-2$,$\frac{n}{2}-3,\;5)$}
**|**, with m = {$\begin{array}{c} \frac{n}{4}-3,\;n\in \left\{12+4k\;/k\in IN\right\}\\ \frac{n}{4}-\frac{7}{2},\;n\in \left\{14+4k\;/k\in IN\right\} \end{array}$
G{n, N = 3, ($\frac{n}{2}-2$,$\frac{n}{2}-2-m,\;4+m)$}${\bar{D}}_{3}$($\frac{1}{\frac{n}{2}-2}<\frac{d}{C}\leq \frac{1}{\frac{n}{2}-3})$G{n, N = 3, ($\frac{n}{2}-3$,$\frac{n}{2}-3,\;6)$}$k_{1}\left(d\right)=\frac{n}{2}-3$G{n, N = 3, ($\frac{n}{2}-3$,$\frac{n}{2}-4,\;7)$}
**|**, with m = {$\begin{array}{c} \frac{n}{4}-\frac{9}{2},\;n\in \left\{18+4k\;/k\in IN\right\}\\ \frac{n}{4}-5,\;n\in \left\{20+4k\;/k\in IN\right\} \end{array}$
G{n, N = 3, ($\frac{n}{2}-3$,$\frac{n}{2}-3-m,\;6+m)$}${\bar{D}}_{q}$($\frac{1}{\frac{n}{2}-q+1}<\frac{d}{C}\leq \frac{1}{\frac{n}{2}-q})$G{n, N = 3, ($\frac{n}{2}-q$,$\frac{n}{2}-q,\;2q)$}$k_{1}\left(d\right)=\frac{n}{2}-q$G{n, N = 3, ($\frac{n}{2}-q$,$\frac{n}{2}-q-1,\;2q+1)$}
**|,** with m = {$\begin{array}{c} \frac{n}{4}-\frac{3q}{2},\;n\in \left\{6q+4k\;/k\in IN\right\}\\ \frac{n}{4}-\frac{\left(3q+1\right)}{2},\;n\in \left\{6q+2+4k\;/k\in IN\right\} \end{array}$
G{n, N = 3, ($\frac{n}{2}-q$,$\frac{n}{2}-q-m,\;2q+m)$}where *q* is the smallest integer value satisfying the following inequality:

$$3\left(\frac{n}{2}-q-1\right)<n$$

It follows that q is given by:

(3) $$q=floor\left(\frac{n}{6}\right)$$

which can be written in the following expression:

$$q=\left\{\begin{array}{c} \frac{n}{6}-\frac{1}{\pi }arccot\left[cot\left(\frac{n\pi }{6}\right)\right],\;n\neq 12k+6/k\in IN\\ \frac{n}{6},\;\;\;\;\;\;\;\;\;\;\;\;\;\;\;\;\;\;\;\;\;\;\;\;\;\;\;\;\;\;\;\;\;\;\;\;\;\;\;n=12k+6/k\in IN \end{array}\right\}$$

We note that for any odd (or even) n, each domain D_i_ (${\bar{D}}_{i})$ corresponds to certain number of equivalent graphs. This later represents the degeneracy of the domain D_i_ (${\bar{D}}_{i})$.

### 5.2. General Expressions of the Domain Degeneracy

The detailed analysis of the domains and their corresponding graphs presented in Section 5.1: (a) and (b) of this section, allow us to establish general expressions of their degeneracy for both odd and even numbers n of vaccination centres. We have indicated that the defined degeneracy deg(n, N, D_ℓ_ (or ${\bar{D}}_{i}$)) depends on four sets covering all possible integer values. Thus,

(i) For n∈ {3+4k/ k∈IN}
  (4) $$\deg(n,\;N=3,\;D_{\ell })=\left\{\begin{array}{c} \frac{n}{4}-\frac{3}{4}\left(2\ell +1\right)+1,\;if\;\ell \;is\;even\\ \frac{n}{4}-\frac{3}{4}\left(2\ell +1\right)+\frac{1}{2},\;if\;\ell \;is\;odd \end{array}\right\}$$
  D_ℓ_ denotes the domain: $\frac{1}{\frac{n+1}{2}-\ell }<\frac{d}{C}\leq \frac{1}{\frac{n-1}{2}-\ell }$
  where
  (5) $$\ell \in \left\{0,1,\;2,\;3,\ldots ,floor\left(\frac{n-9}{6}\right)+1\right\}$$

(ii) For n∈ {5+4k/ k∈IN}
  (6) $$\deg(n,\;N=3,\;D_{\ell })=\left\{\begin{array}{c} \frac{n}{4}-\frac{3}{4}\left(2\ell +1\right)+1,\;if\;\ell \;is\;odd\\ \frac{n}{4}-\frac{3}{4}\left(2\ell +1\right)+\frac{1}{2},\;if\;\ell \;is\;even \end{array}\right\}$$
  D_ℓ_ denotes the domain: $\frac{1}{\frac{n+1}{2}-\ell }<\frac{d}{C}\leq \frac{1}{\frac{n-1}{2}-\ell }$
  where $\ell \in \left\{0,1,\;2,\;3,\ldots ,floor\left(\frac{n-9}{6}\right)+1\right\}$.

**Figure 5:**
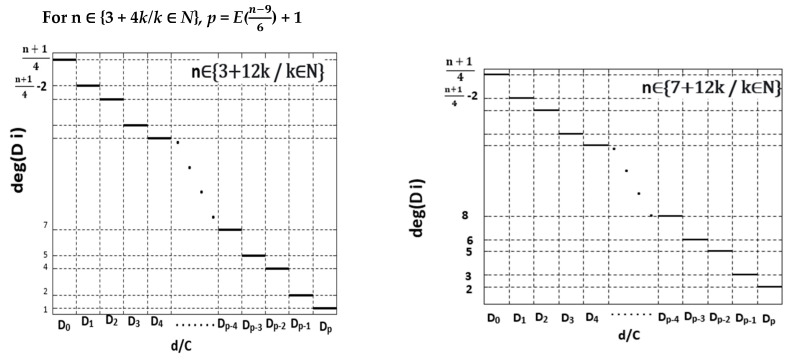

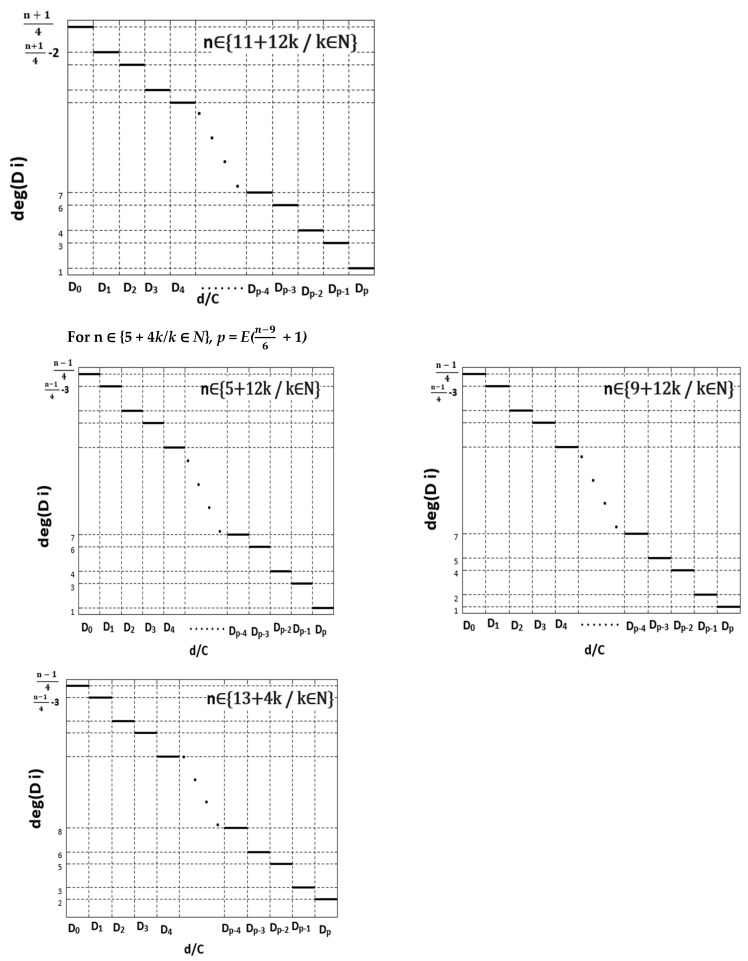
A generalization making possible to find the variation of domain degeneracy for any odd number of vaccination centres.

In Appendix B, we represent the variation of domain degeneracy as a function of vaccination centre demands «site needs» for selected odd values of n. Furthermore, we plot in Figure 5, a generalization making possible to find the variation of such degeneracy for any odd number of vaccination centres.

(i) For n∈ {6+4k/ k∈IN}
  (7) $$\deg(n,N=3,\;{\bar{D}}_{\ell })=\left\{\begin{array}{c} \frac{n}{4}-\frac{3\ell }{2}+1,\;if\;\ell \;is\;odd\\ \frac{n}{4}-\frac{\left(3\ell +1\right)}{2}+1,\;if\;\ell \;is\;even \end{array}\right\}$$
  $\bar{D}$_ℓ_ denotes the domain: $\frac{1}{\frac{n}{2}-\ell +1}<\frac{d}{C}\leq \frac{1}{\frac{n}{2}-\ell }$
  where
  (8) $$\ell \in \left\{1,\;2,\;3,\ldots ,floor\left(\frac{n}{6}\right)\right\}$$

(ii) For n∈ {8+4k/ k∈IN}
  (9) $$\deg(n,N=3,\;{\bar{D}}_{\ell })=\left\{\begin{array}{c} \frac{n}{4}-\frac{3\ell }{2}+1,\;if\;\ell \;is\;even\\ \frac{n}{4}-\frac{\left(3\ell +1\right)}{2}+1,\;if\;\ell \;is\;odd \end{array}\right\}$$
  $\bar{D}$_ℓ_ denotes the domain: $\frac{1}{\frac{n}{2}-\ell +1}<\frac{d}{C}\leq \frac{1}{\frac{n}{2}-\ell }$
  where $\ell \in \left\{1,\;2,\;3,\ldots ,floor\left(\frac{n}{6}\right)\right\}$.

**Figure 6:**
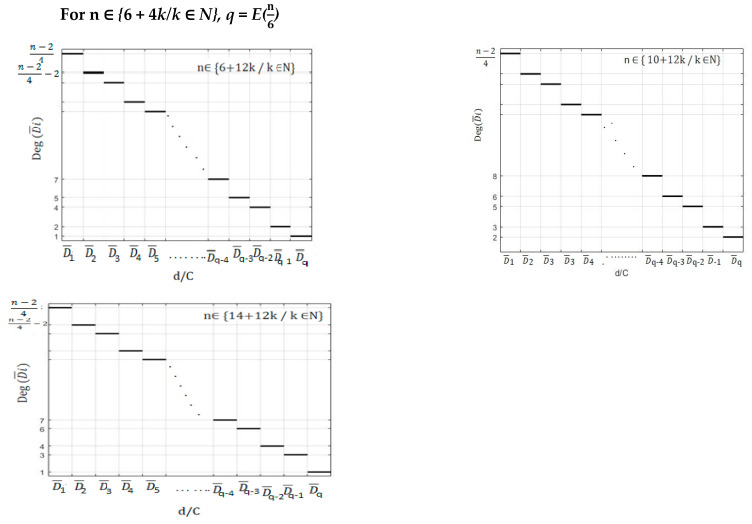

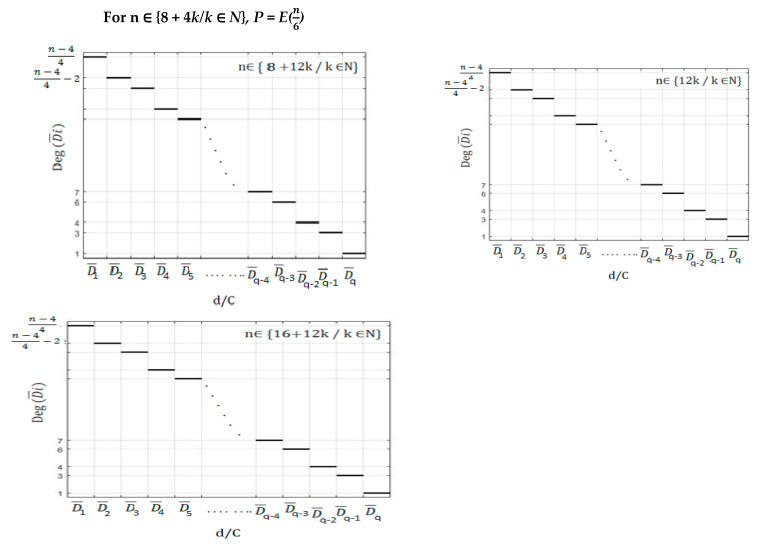
A generalization making possible to find the variation of domain degeneracy for any even number of vaccination centres.

In Appendix C, we represent the variation of domain degeneracy as a function of the vaccination centres demands for selected even values of n. Furthermore, we plot in Figure 6, a generalization making possible to find the variation of such degeneracy for any even number of vaccination centres.

As is observed in Appendixe B and Appendixe C and in Figure 5 and Figure 6, these variations are represented by what we can call “descending ceiling functions”.

**Figure 7:**
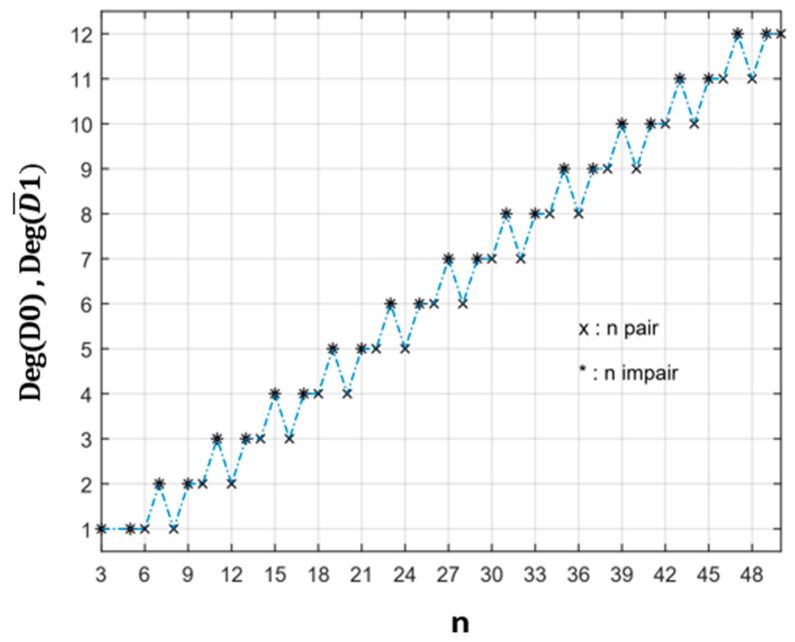
Variation of degree of degeneracy deg(D_0_) and deg($\bar{D}$_1_) with n.

In Figure 7, we reported the variation of the degrees of degeneracy deg(D_0_) and deg($\bar{D}$_1_). They correspond to the most degenerate domains for odd and even numbers of vaccination centres.

**Figure 8:**
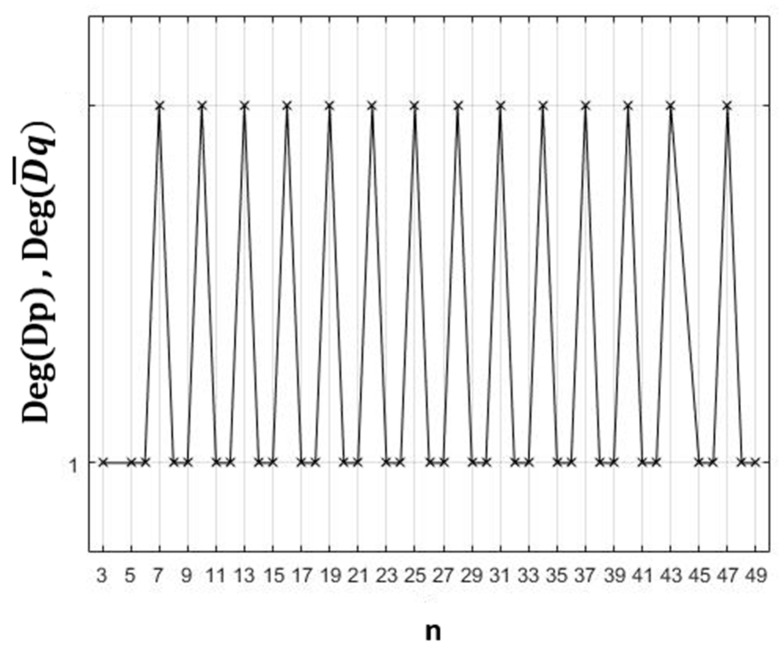
Variation of the degrees of degeneracy deg(D_p_) and deg($\bar{D}$_q_) corresponding to the least degenerate domains.

On the other hand, in Figure 8, we plot the variation of the degrees of degeneracy deg(D_p_) and deg($\bar{D}$_q_) corresponding to the least degenerate domains for odd n and even n, respectively. They can be expressed by the following functions:

$$\deg(D_{p})=1\mathrm{or}\deg({\bar{D}}_{q})=1,\;\mathrm{if}\;n\in \left\{3k/k\in IN^{*}\right\}\cup \left\{5+3k/k\in IN\right\},$$

$$\deg(D_{p})=2\mathrm{or}\deg({\bar{D}}_{q})=2,\;\mathrm{if}\;n\;\in \{7+3k/k\in IN\},$$

where *p* = $floor\left(\frac{n-9}{6}\right)+1,$ and *q* = $floor\left(\frac{n}{6}\right)$.

### 5.3. General Expressions of the Number of Graphs (Different Paths) Using an Optimum Number N of Drones for Logistics Distribution

Let us first note that for the trivially case, namely $\frac{d}{C}\leq \frac{1}{n}$, delivery can be realized only by one drone. In this case, it is obvious that the drone has only one path to follow to accomplish the task, and therefore there exist only one graph which can be noted G{n, N = 1,(n)}, according to the adopted notation.

(a) General expressions of the number of graphs (paths) when the optimal number of drones is 2 (N = 2)

In the case N = 2 as optimum number of drones satisfying the maximum vaccination centres according to their demands, the corresponding domains and graphs are defined by the following intervals:
(i) For odd n
Domain Corresponding graph$D_{1}$($\frac{1}{n}<\frac{d}{C}\leq \frac{1}{n-1})$G{n, N = 2, (n−1,1)}$D_{2}$($\frac{1}{n-1}<\frac{d}{C}\leq \frac{1}{n-2})$G{n, N = 2, (n−2,2)}$D_{3}$($\frac{1}{n-2}<\frac{d}{C}\leq \frac{1}{n-3})$G{n, N = 2, (n−3,3)}$D_{\frac{n-1}{2}}$($\frac{1}{\frac{n+3}{2}}<\frac{d}{C}\leq \frac{1}{\frac{n+1}{2}})$G{n, N = 2, ($\frac{n+1}{2},\;\frac{n-1}{2})$}(ii) For even n
Domain Corresponding graph${\bar{D}}_{1}$($\frac{1}{n}<\frac{d}{C}\leq \frac{1}{n-1})$G{n, N = 2, (n−1,1)}${\bar{D}}_{2}$($\frac{1}{n-1}<\frac{d}{C}\leq \frac{1}{n-2})$G{n, N = 2, (n−2,2)}${\bar{D}}_{3}$($\frac{1}{n-2}<\frac{d}{C}\leq \frac{1}{n-3})$G{n, N = 2, (n−3,3)}${\bar{D}}_{\frac{n}{2}}$($\frac{1}{\frac{n}{2}+1)}<\frac{d}{C}\leq \frac{1}{\frac{n}{2}})$G{n, N = 2, ($\frac{n}{2},\;\frac{n}{2})$}

For *N* = 2, each domain *D_i_* or ${\bar{D}}_{i}$ corresponds to one and only one graph. This means that the domains are not degenerated for any number n of vaccination centres and for any demands using necessary two drones. We have to note that, in this case and for a given zone with n centres, all graphs constructed for any domain have the same length *L*(n, N = 2). This later is given by:

(10) $$L\left(n,N=2\right)=\left(2n-7\right)\left[4+2\left(n-2\right)\sin\left(\frac{\pi }{n}\right)\right]r.$$

Therefore, it does not depend on the topology of the graph and the vaccination centre demands when this later belongs to the ranges:

(i) $\left[\frac{C}{n},\frac{C}{\frac{n+1}{2}}\right]$ for odd n

(ii) $\left[\frac{C}{n},\frac{C}{\frac{n}{2}}\right]$ for even n.

**Figure 9:**
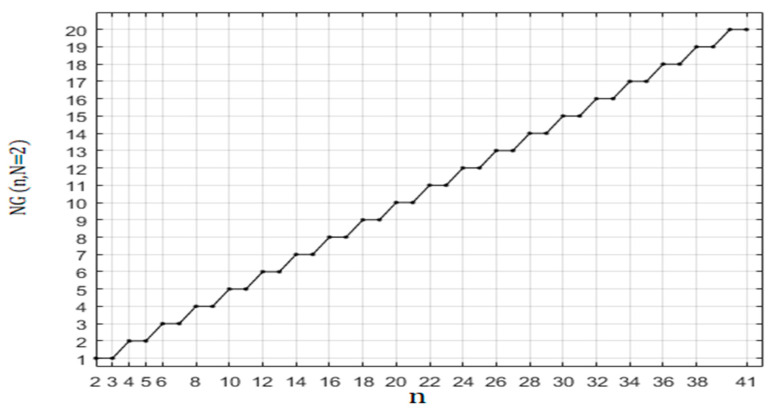
Dependence of the number of graphs *NG* (n, N = 2) as a function of n.

From the above ranges, we deduce that the total number *NG* (*n, N* = 2) of graphs, which correspond to N = 2 and for any number n of vaccination centres belonging to the same zone, is given by:

(11a) $$NG\left(n,\;N=2\right)=\frac{n-1}{2},\mathrm{for}\;\mathrm{odd}\;n$$

(11b) $$NG\left(n,\;N=2\right)=\frac{n}{2},\mathrm{for}\;\mathrm{even}\;n$$

which is represented in Figure 9.

(b) General expressions of the number of graphs (paths) when the optimal number of drones is 3 (N = 3)

Let us first note that when we have to use three UAVs to deliver vaccines to vaccination centres belonging to same zone, all graphs constructed for any domain D_i_ or ${\bar{D}}_{i}$ have the same length which is given by:

(11c) $$L\left(n,N=3\right)=\left(2n-7\right)\left[6+2\left(n-3\right)\sin\left(\frac{\pi }{n}\right)\right]r.$$

Thus, it does not depend on the structure of the graph. As demonstrated in Section 4.1, in order to satisfy the vaccination centres with N = 3 as optimum number of drones and according to our assumptions, the centre demands d have to belong to the following ranges:

(i) $\left[\frac{C}{\frac{n+1}{2}},\frac{C}{\frac{n-1}{2}-p}\right]$, for odd n≥3, with $p=floor\left(\frac{n-9}{6}\right)$+1

(ii) $\left[\frac{C}{\frac{n}{2}},\frac{C}{\frac{n}{2}-q}\right]$, for even n≥6, with $q=floor\left(\frac{n}{6}\right)$.

So, the total number *NG* (n, N = 3) of graphs corresponding to N = 3 depends on the parity of the number n of vaccination centres. Thus,

- For odd n

$$NG\left(n,\;N=3\right)=\overset{p}{\underset{\ell =0}{\sum }}\deg\left(n,\;N=3,\;D_{\ell }\right)$$

Using expressions Equations (4)–(6), we have demonstrated that *NG*(n, N *= 3)* also depends on the parity of *p*. Its expression is given by:

- $\left\{\begin{array}{c} n\in \left\{2k+1/\;k\in IN\right\}\\ floor\left(\frac{n-9}{6}\right)+1,\;\mathrm{odd}\; \end{array}\right\}$,

(12) $$NG\left(n,\;N=3\right)=\frac{1}{4}\left[floor\left(\frac{n-9}{6}\right)+2\right]\left[n-3floor\left(\frac{n-9}{6}\right)-3\right].$$

- $\left\{\begin{array}{c} n\in \left\{3+4k/\;k\in IN\right\}\\ floor\left(\frac{n-9}{6}\right)+1,\;\mathrm{even} \end{array}\right\}$,

(13) $$NG\left(n,\;N=3\right)=\left[floor\left(\frac{n-9}{6}\right)+2\right]\left[\frac{n}{4}+1\right]-\frac{1}{4}\left[floor\left(\frac{n-9}{6}\right)+1\right]\left[3floor\left(\frac{n-9}{6}\right)+10\right]-\frac{3}{4}.$$

- $\left\{\begin{array}{c} n\in \left\{5+4k/\;k\in IN\right\}\\ floor\left(\frac{n-9}{6}\right)+1,\;\mathrm{even} \end{array}\right\}$,

(14) $$NG\left(n,\;N=3\right)=\frac{1}{4}\left[floor\left(\frac{n-9}{6}\right)+2\right]\left[n-3floor\left(\frac{n-9}{6}\right)-3\right]-\frac{1}{4}.$$

**Figure 10:**
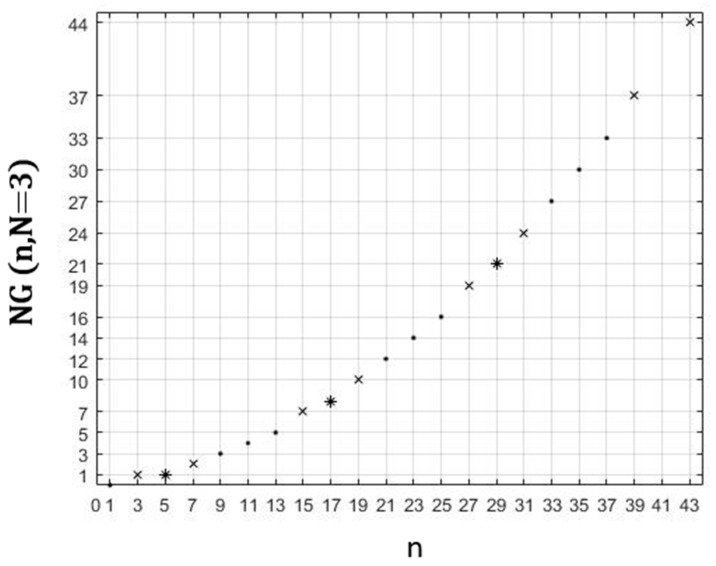
Dependence of the number of graphs *NG*(n, N = 3) as a function of odd n. **•**: (n∈{2k+1/ k∈IN}, odd p); **x**: (n∈{3+4k/ k∈IN}, even p); *: (n∈{5+4k/k∈IN}, even p).

In Figure 10, we represent the evolution of the number of graphs *NG*(n, N = 3) in zones containing odd numbers of vaccination centres.

- For even n

$$NG\left(n,\;N=3\right)=\overset{q}{\underset{\ell =1}{\sum }}\deg\left(n,\;N=3,\;{\bar{D}}_{\ell }\right)$$

Using Equations (7)–(9), we have demonstrated that *NG*(n, N = 3) also depends on the parity of q. Its expression is given by:

(iii) $\left\{\begin{array}{c} n\in \left\{2k/\;k\in IN\right\}\\ floor\left(\frac{n}{6}\right)\;\mathrm{even}\; \end{array}\right\}$,
  (15) $$NG\left(n,\;N=3\right)=\frac{n}{4}\left[floor\left(\frac{n}{6}\right)\right]-\frac{1}{4}floor\left(\frac{n}{6}\right)\left[3floor\left(\frac{n}{6}\right)+4\right]+floor\left(\frac{n}{6}\right)$$

(iv) $\left\{\begin{array}{c} n\in \left\{6+4k/\;k\in IN\right\}\\ floor\left(\frac{n}{6}\right),\;\mathrm{odd}\; \end{array}\right\}$,
  (16) $$NG\left(n,\;N=3\right)=\frac{n}{4}\left[floor\left(\frac{n}{6}\right)\right]-\frac{1}{4}\left[floor\left(\frac{n}{6}\right)-1\right]\left[3floor\left(\frac{n}{6}\right)+1\right]-\frac{1}{2}floor\left(\frac{n}{6}\right)$$

(v) $\left\{\begin{array}{c} n\in \left\{8+4k/\;k\in IN\right\}\\ floor\left(\frac{n}{6}\right),\;\mathrm{odd}\; \end{array}\right\}$,
  (17) $$NG\left(n,\;N=3\right)=\frac{n}{4}\left[floor\left(\frac{n}{6}\right)\right]-\frac{1}{4}\left[3floor\left(\frac{n}{6}\right)+1\right]\left[floor\left(\frac{n}{6}\right)+1\right]+floor\left(\frac{n}{6}\right)$$

**Figure 11:**
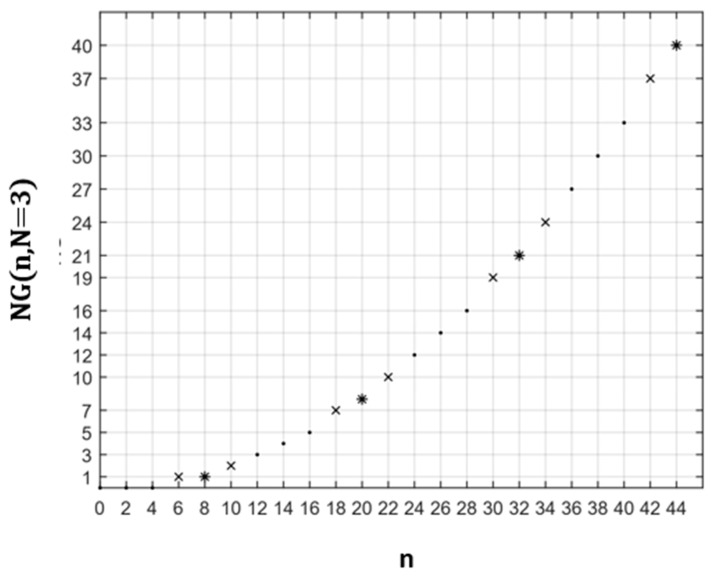
Dependence of the number of graphs *NG*(n, N = 3) as a function of even n. **•**: (n∈{2k/ k∈IN}, even q); **x**: (n∈{6+4k/ k∈IN}, odd q); *: (n ∈{8+4k/k∈IN}, odd q).

In Figure 11, we plot the variation of the number of graphs *NG*(n, N = 3) in zones containing even numbers of vaccination centres.

## 6. Conclusions

Over the last years, the use of drones is leading to new working models in a variety of logistic scenarios. This happens in both developed and developing countries. Currently, fascinating pilot and implementation projects, especially in Africa and Asia, reveal their true potential. By some predictions, the commercial drone market will triple by 2023.In healthcare section, the potential for drone use is vast, and hopefully drones are able to fill some niches where our performance needs improvement. Drones may significantly increase access to healthcare for individuals and large populations, particularly those who do not benefit from appropriate care due to remoteness and lack of infrastructure or funds. In this paper, we have proposed an exact analytical approach and operational strategy to the logistics of any pandemic vaccination effort. Using Unmanned Aerials vehicles (drones), our approach developed a procedure which can be used to deliver pandemic vaccines to densely populated cities or sparsely populate rural areas, whatever the number of population living there. Our strategy consists of dividing the concerned territorial area into zones in the form of circular bands. Each zone is divided into several isometric districts where each of them contains a vaccination centre. We have been interested in the route planning problem of the drones during the distribution. This latter concern the vaccines delivery from central depot to any number of vaccination centres. In the case of fixed and optimal number of the used drones, we have determined all equivalent and optimal flight path plans (graphs). This depends on the domain D of vaccination centre demand d. The existence of equivalent graphs, corresponding to a well-known domain d, incites us to define what we called “degeneracy of the domain Dd”. The equivalent graphs (paths) have de same length, the same global cost and the same delivery time. It is worthy to note that, the existence of such degeneracy gives us the possibility to choose the most appropriate paths according to the emergency states of the vaccination centres. Then, using the notion, of degeneracy of the domain D for all domains, we have calculated the total number of graphs (paths) corresponding to the fixed and optimal number of the used drones. In our present investigation all relations has been expressed for any number of vaccination centres.

## Figures and Tables

**Table 1:** Domains of d, corresponding graphs and lengths *L* (*n, N*) of the paths travelled by the set of optimum number of drones necessary for the logistics distribution, when zone contains n = 7, 8, and 9 vaccination centres, respectively.

n = 7				
d/C	$k_{1}\left(d\right)$	N	Graphs	L(n,N)
$\frac{d}{C}\leq \frac{1}{7}$	7	1	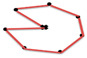 G = {n = 7, N = 1, (7)}	50.446 r
$\frac{1}{7}<\frac{d}{C}\leq \frac{1}{6}$	6	2	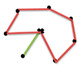 G = {n = 7, N = 2, (6, 1)}	58.371 r
$\frac{1}{6}<\frac{d}{C}\leq \frac{1}{5}$	5	2	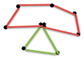 G = {n = 7, N = 2, (5, 2)}	
$\frac{1}{5}<\frac{d}{C}\leq \frac{1}{4}$	4	2	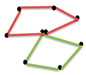 G = {n = 7, N = 2, (4, 3)}	
$\frac{1}{4}<\frac{d}{C}\leq \frac{1}{3}$	3	3	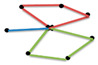 G = {n = 7, N = 3, (3, 2, 2)} 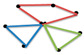 G = {n = 7, N = 3, (3, 3, 1)}	66.297 r
$\frac{1}{3}<\frac{d}{C}\leq \frac{1}{2}$	2	4	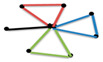 G = {n = 7, N = 4, (2, 2, 2, 1)}	74.223 r
$\frac{1}{2}<\frac{d}{C}\leq 1$	1	7	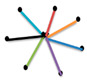 G = {n = 7, N = 7, (1, 1, 1, 1, 1, 1, 1)}	98 r
n = 8				
d/C	$k_{1}\left(d\right)$	N	Graphs	L(n,N)
$\frac{d}{C}\leq \frac{1}{8}$	8	1	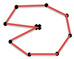 G = {n = 8, N = 1, (8)}	66.218 r
$\frac{1}{8}<\frac{d}{C}\leq \frac{1}{7}$	7	2	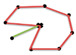 G = {n = 8, N = 2, (7, 1)}	77.329 r
$\frac{1}{7}<\frac{d}{C}\leq \frac{1}{6}$	6	2	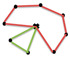	
$\frac{1}{6}<\frac{d}{C}\leq \frac{1}{5}$	5	2	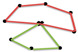	
$\frac{1}{5}<\frac{d}{C}\leq \frac{1}{4}$	4	2	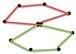	
$\frac{1}{4}<\frac{d}{C}\leq \frac{1}{3}$	3	3	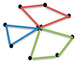	88.441 r
$\frac{1}{3}<\frac{d}{C}\leq \frac{1}{2}$	2	4	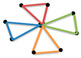 G = {n = 8, N = 4, (2, 2, 2, 2)}	99.553 r
$\frac{1}{2}<\frac{d}{C}\leq 1$	1	8	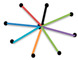 G = {n = 8, N = 8, (1, 1, 1, 1, 1, 1, 1, 1)}	144 r
n = 9				
d/C	$k_{1}\left(d\right)$	N	Graphs	L(n,N)
$\frac{d}{C}\leq \frac{1}{9}$	9	1	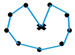 G = {n = 9, N = 1, (9)}	82.195 r
$\frac{1}{9}<\frac{d}{C}\leq \frac{1}{8}$	8	2	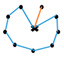 G = {n = 9, N = 2, (8, 1)}	96.6711 r
$\frac{1}{8}<\frac{d}{C}\leq \frac{1}{7}$	7	2	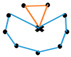 G = {n = 9, N = 2, (7, 2)}	
$\frac{1}{7}<\frac{d}{C}\leq \frac{1}{6}$	6	2	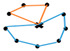 G = {n = 9, N = 2, (6, 3)}	
$\frac{1}{6}<\frac{d}{C}\leq \frac{1}{5}$	5	2	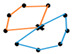 G = {n = 9, N = 2, (5, 4)}	
$\frac{1}{5}<\frac{d}{C}\leq \frac{1}{4}$	4	3	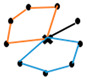 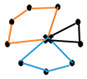 G = {n = 9, N = 3, (4, 4, 1)} G = {n = 9, N = 3, (4, 3, 2)}	111.146 r
$\frac{1}{4}<\frac{d}{C}\leq \frac{1}{3}$	3	3	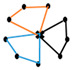 G = {n = 9, N = 3, (3, 3, 3)}	
$\frac{1}{3}<\frac{d}{C}\leq \frac{1}{2}$	2	5	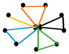 G = {n = 9, N = 5, (2, 2, 2, 2, 1)}	140.097 r
$\frac{1}{2}<\frac{d}{C}\leq 1$	1	9	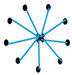 G = {n = 9, N = 9, (1, 1, 1, 1, 1, 1, 1, 1, 1)}	198 r

**Figure A1:**
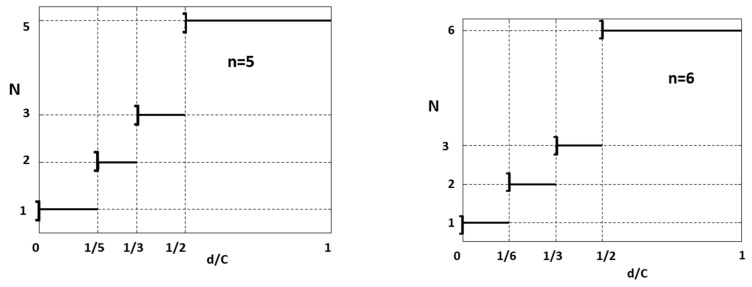
The variation of optimum number N of drones as function of vaccination centre demands, for n = 5, and 6.

**Figure A2:**
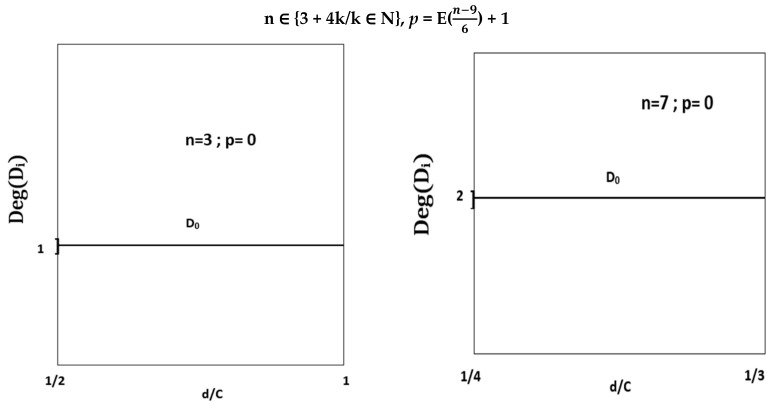

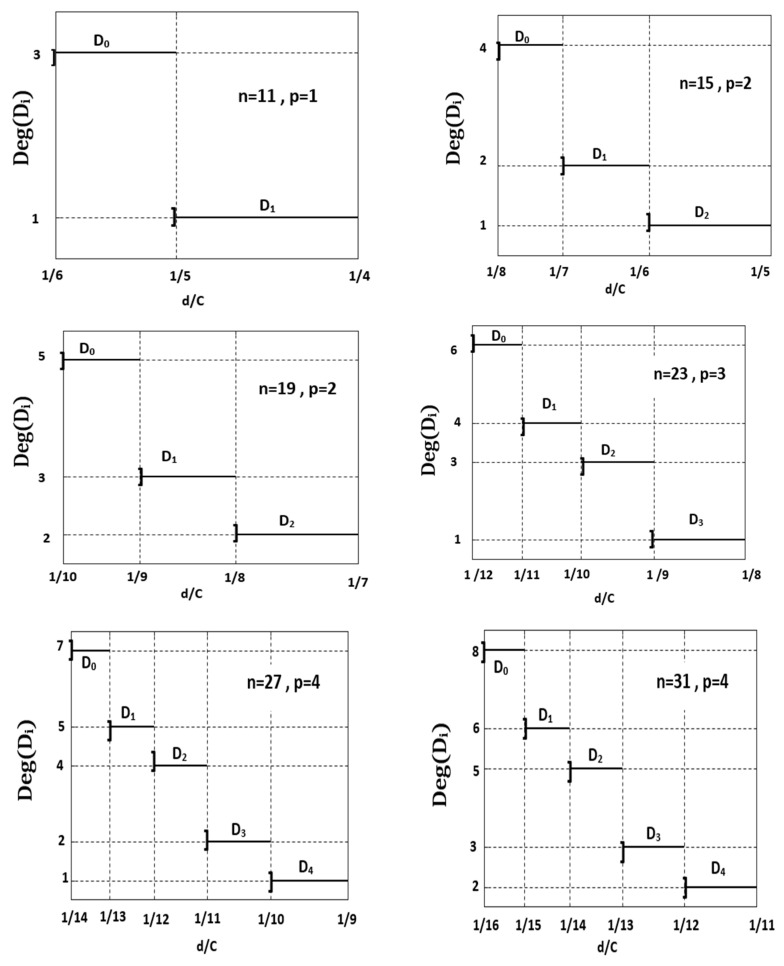

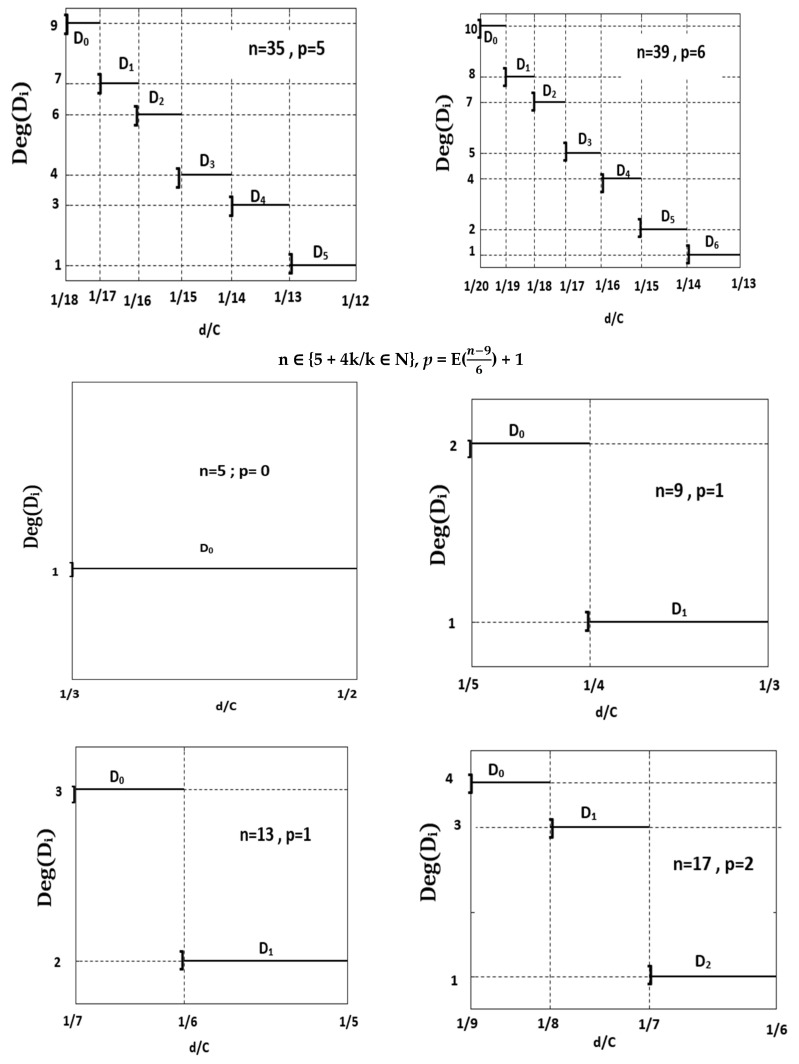

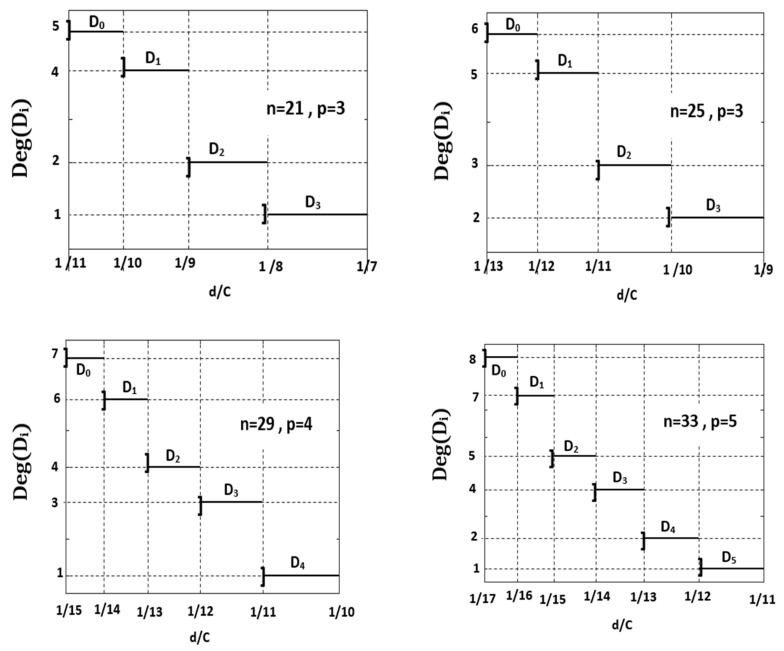
The variation of domain degeneracy as a function of vaccination centre demands «site needs» for selected odd values of n.

**Figure A3:**
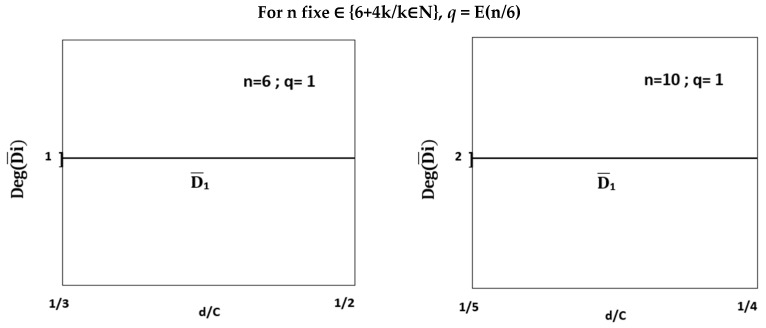

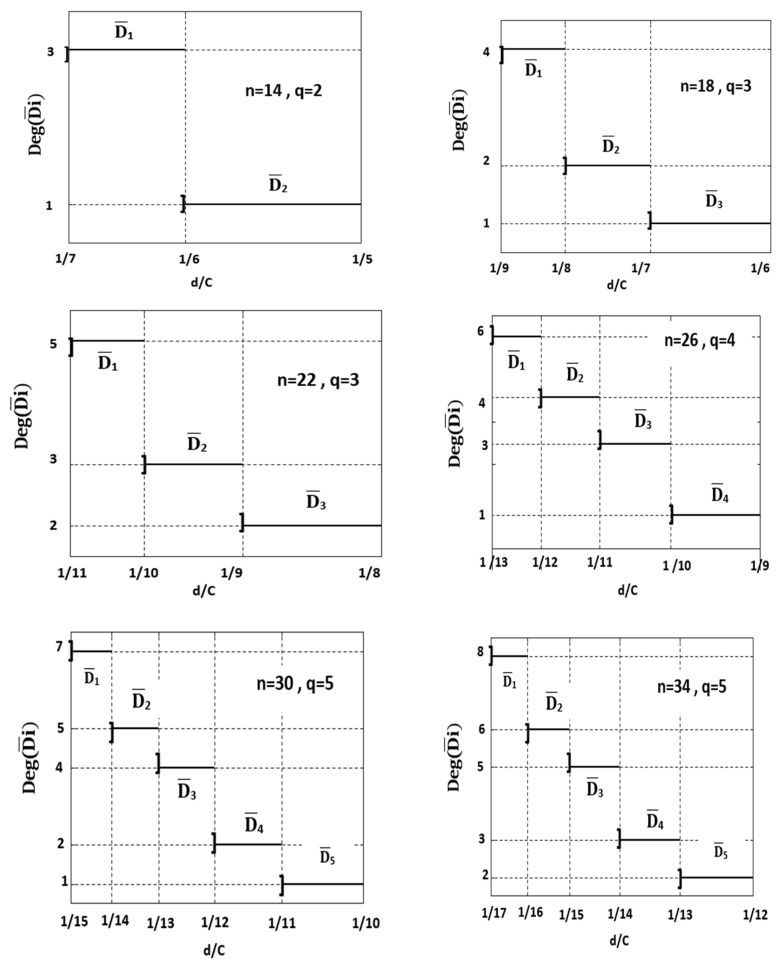

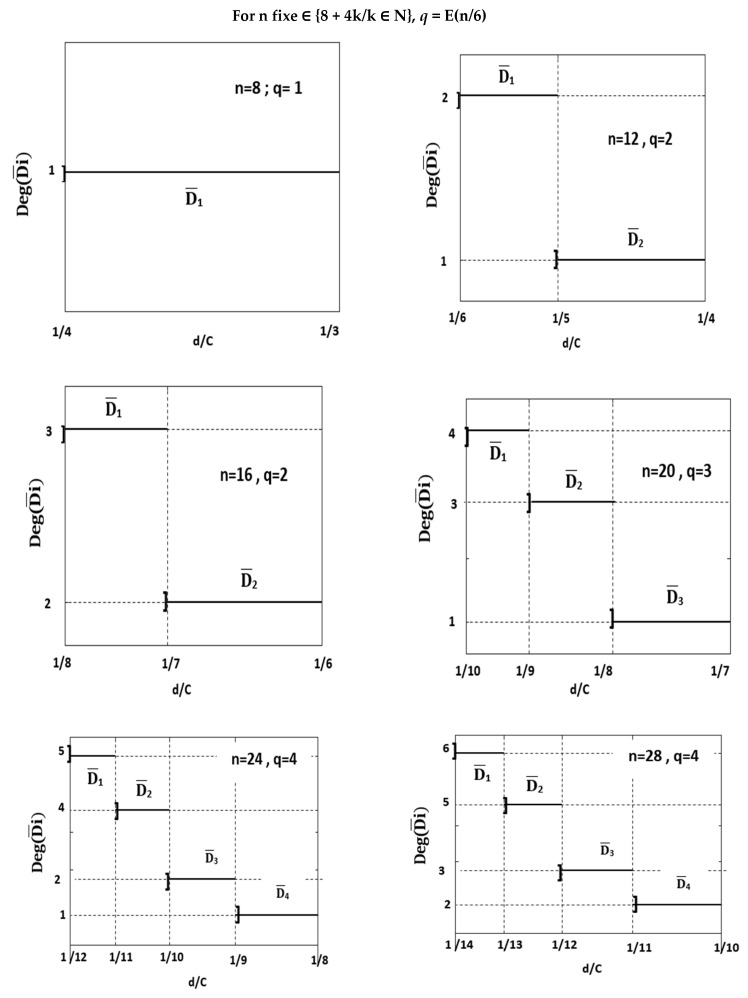

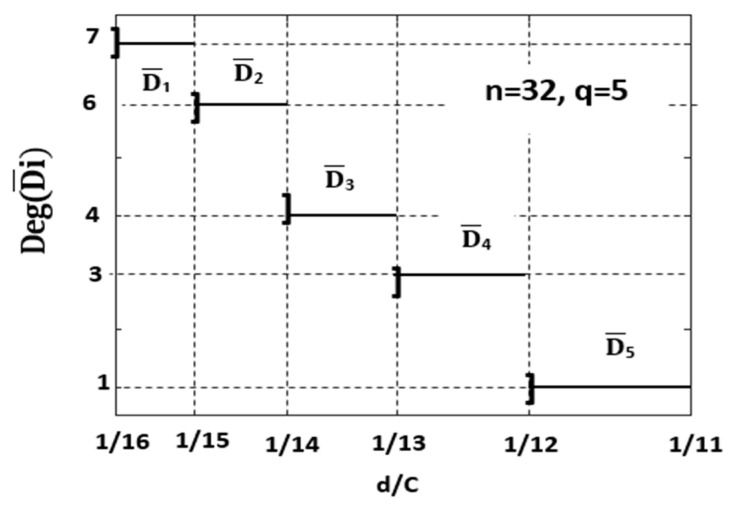
The variation of domain degeneracy as a function of the vaccination centres demands for selected even values of n.

## Data Availability

Not applicable.

## Appendix A

**Table A1:** Domains of d, corresponding graphs and lengths *L* (n, N) of the paths travelled by the set of optimum number of drones necessary for the logistics distribution, when zone contains n = 5, and 6 vaccination centres, respectively.

n = 5				
d/C	$k_{1}\left(d\right)$	N	Graphs	L(n,N)
$\frac{d}{C}\leq \frac{1}{5}$	5	1	G = {n = 5, N = 1, (5)}	20.106 r
$\frac{1}{5}<\frac{d}{C}\leq \frac{1}{4}$	4	2	G = {n = 5, N = 2, (4, 1)}	22.580 r
$\frac{1}{4}<\frac{d}{C}\leq \frac{1}{3}$	3	2	G = {n = 5, N = 2, (3, 2)}	
$\frac{1}{3}<\frac{d}{C}\leq \frac{1}{2}$	2	3	G={n = 5, N = 3, (2, 2, 1)}	25.053 r
$\frac{1}{2}<\frac{d}{C}\leq 1$	1	5	G = {n = 5, N = 5, (1, 1, 1, 1, 1)}	30 r
n = 6				
d/C	$k_{1}\left(d\right)$	N	Graphs	L(n,N)
$\frac{d}{C}\leq \frac{1}{6}$	6	1	G = {n = 6, N = 1,(6)}	35 r
$\frac{1}{6}<\frac{d}{C}\leq \frac{1}{5}$	5	2	G = {n = 6,N = 2, (5, 1)}	40 r
$\frac{1}{5}<\frac{d}{C}\leq \frac{1}{4}$	4	2	G = {n = 6, N = 2, (4, 2)}	
$\frac{1}{4}<\frac{d}{C}\leq \frac{1}{3}$	3	2	G = {n = 6, N = 2, (3, 3)}	
$\frac{1}{3}<\frac{d}{C}\leq \frac{1}{2}$	2	3	G = {n = 6, N = 3, (2, 2, 2)}	45 r
$\frac{1}{2}<\frac{d}{C}\leq 1$	1	6	G = {n = 6, N = 6, (1, 1, 1, 1, 1, 1)}	60 r
